# Rapid Titration of Methadone for Opioid Use Disorder in the Emergency Department: A Case Report

**DOI:** 10.5811/cpcem.39968

**Published:** 2025-03-20

**Authors:** Miles Lamberson, Roz King, Colin T. Waters, Peter Jackson, John Brooklyn, Elly Riser, Daniel Wolfson

**Affiliations:** * University of Vermont Larner College of Medicine, Department of Emergency Medicine, Burlington, Vermont University of Vermont; † Larner College of Medicine, Department of Psychiatry, Burlington, Vermont; ‡ University of Vermont, Vermont Center for Behavior and Health, Center on Rural Addiction, Burlington, Vermont; § University of Vermont Larner College of Medicine, Department of Family Medicine, Burlington, Vermont; ¶ University of Vermont Larner College of Medicine, Department of Medicine, Burlington, Vermont

**Keywords:** case report, methadone, rapid titration, medications for opioid use disorder, emergency department

## Abstract

**Introduction:**

The prevalence of high-potency synthetic opioids (HPSO), such as fentanyl and its analogs, presents significant treatment challenges to current strategies for emergency department (ED) initiation of medication for opioid use disorder (MOUD). While most EDs traditionally use buprenorphine for MOUD, its effectiveness can be limited in patients exposed to HPSOs due to risk of precipitated withdrawal or inadequate control of withdrawal symptoms. Methadone, a full agonist, is another MOUD agent that addresses severe withdrawal symptoms and cravings associated with HPSO dependence and will not cause precipitated withdrawal. Traditional methadone protocols often fail to provide sufficient doses to alleviate withdrawal symptoms, but new federal guidelines allow higher initial doses and rapid titration to therapeutic levels.

**Case Report:**

We report a case of rapid methadone titration in the ED for a patient with a history of high HPSO utilization. The patient received an initial dose of methadone 50 milligrams (mg) orally, followed by titration of additional 10 mg doses hourly to a cumulative dose of 70 mg at discharge. Vital signs, mental status, and Clinical Opiate Withdrawal Scale scores were monitored to guide dosing.

**Conclusion:**

The protocol allowed for safe, individualized care, achieving therapeutic dosing levels that alleviated withdrawal symptoms and enabled the patient to transition to outpatient follow-up treatment. This approach addresses the need for rapid, effective methadone initiation in an era in which high-potency synthetic opioids pose challenges to traditional opioid use disorder treatment.

## INTRODUCTION

The rise in high-potency synthetic opioids (HPSO), including fentanyl and its analogs, is the leading cause of drug overdose deaths in the United States.^1^ Emergency department (ED) initiation of buprenorphine for patients with opioid use disorder (OUD) has proven effective in enhancing medication for opioid use disorder (MOUD) treatment engagement and reducing illicit opioid use.^2^,^3^ However, HPSOs present new treatment challenges due to their lipophilic nature, which leads to accumulation in adipose tissue and heightens the risk of precipitated withdrawal when initiating buprenorphine, a high-affinity partial m-opioid receptor agonist that displaces other opioids including fentanyl.^4^ Methadone, a full m-opioid receptor agonist, is advantageous in patients using HPSOs as it does not precipitate withdrawal.^4^

Patients who struggle to stop using HPSOs due to withdrawal intolerance or who have experienced precipitated withdrawal with buprenorphine often show poor MOUD retention, hesitancy to retry buprenorphine, and a preference for treatment with methadone.^5^ However, methadone has been underused in the ED due to concerns for respiratory depression and restrictive federal regulations.^6^ Until recently, the US Substance Abuse and Mental Health Services Administration guidelines for methadone recommended a maximum initial dose of 30 milligrams (mg). Subsequent dose increases by 5 to 10 mg every 3–5 days often required weeks of titration to achieve therapeutic levels.^7^ This dosing regimen was established before the era of HPSOs. Patients often require maintenance doses of 120 mg/day or greater.^4^,^8^ In the current HPSO era, there is a critical need to develop methadone titration protocols that achieve a therapeutic dose more quickly, increasing treatment retention and reducing the risk of fatal overdose.^9^

Recent changes in 42 Code of Federal Regulations Part 8 allow for higher initial dosing of methadone (up to 50 mg) with additional dosing at the physician’s discretion if a clinical indication (eg, persistent withdrawal symptoms) is documented.^10^ We previously described the feasibility of methadone initiation in the ED for patients with OUD through our Start Treatment and Recovery (STAR) program.^11^ In this case report, we present modifications to our protocol that enabled the safe and rapid titration of methadone to therapeutic levels in the ED for a patient with OUD.

## CASE REPORT

With written patient consent, we report on a 34-year-old male who presented to the ED seeking inpatient treatment for his OUD. The patient reported a history of severe OUD using fentanyl six times daily via intravenous (IV) injection. Upon arriving at the ED in the early morning, the patient showed no signs of withdrawal, had normal vital signs and no other complaints, but he appeared sedated, consistent with recent opioid utilization. A peer recovery coach (PRC) was paged to assist in identifying residential treatment programs. The patient slept most of the day while the PRC explored placement options. Upon awakening, the patient reported withdrawal symptoms, including restlessness, sweating, and agitation, and expressed interest in MOUD.

Using shared decision-making, the emergency physician and STAR coordinator had a detailed conversation with the patient regarding his choice of MOUD with either buprenorphine/naloxone or methadone. Given the patient’s use of HPSOs and prior precipitated withdrawal with buprenorphine, methadone was chosen. The patient recognized that his chances of success in a residential treatment program would improve if he were first stabilized on an effective dose of MOUD. He agreed to initiate methadone in the ED, titrate the dose as much as possible per protocol, and follow up with our local, affiliated outpatient treatment program (OTP) for further dose adjustments before enrolling in a residential program.

**CPC-EM Capsule**

What do we already know about this clinical entity?

*Buprenorphine may precipitate withdrawal. Methadone is an alternative, but traditional dosing may not adequately control symptoms*.

What makes this presentation of disease reportable?

*We describe rapid methadone titration in the emergency department (ED), safely initiating treatment at 50 mg and titrating to a cumulative 70 mg dose to manage withdrawal symptoms*.

What is the major learning point?

*New federal guidelines permit an increase in the initial methadone dose from 30 to 50 mg, with further titration to higher doses when clinically indicated*.

How might this improve emergency medicine practice?

*Given the prevalence of high-potency synthetic opioids, increasing methadone doses can improve withdrawal management and retention in treatment for opioid use disorder*.

The patient was treated using our rapid methadone titration pathway, which includes an initial dose of 40–50 mg orally based on clinician discretion, followed by 10 mg every hour, as clinically indicated for persistent withdrawal symptoms, until symptomatic relief is achieved, or a maximum dose of 70 mg is reached. Titrated dosing is only provided if the patient’s Clinical Opiate Withdrawal Scale (COWS) score is greater than five, and there are no signs of sedation including hypoventilation (SpO_2_ less than 94% on room air or respiratory rate less than 12 respirations per minute) or hypotension (systolic blood pressure less than 100 millimeters of mercury). Exclusion criteria are considered prior to rapid methadone titration. Contraindications include known QTc prolongation and medical comorbidities such as pulmonary disease, cirrhosis, end-stage renal disease, congestive heart failure, or ventricular arrhythmia. Relative contraindications include the concurrent prescription of benzodiazepines, tricyclic antidepressants, alcohol or other sedatives, and medications affecting methadone metabolism (eg, CYP inhibitors and inducers) (Figure).^9^

At the time of MOUD initiation the patient had a COWS score of 14, and 50 mg of methadone was administered. The patient was reassessed after one hour. He had no signs of sedation or respiratory depression, and the recalculated COWS score was 10. An additional 10 mg of methadone was given. Another 10 mg methadone dose was administered 90 minutes later for persistent withdrawal symptoms and a COWS score of 6, for a total dose of 70 mg. Five hours after the initial dose, the patient again had a COWS score of 6 but stated that he felt improved. At no point were any complications observed. No additional medications were administered during the ED visit. The patient was observed for one hour after the final methadone dose, prior to discharge (Table). The patient successfully followed up with the outpatient treatment program (OTP) the next morning at which time he was given an 80 mg dose of methadone. This was followed by an additional 100 mg dose the following day, both administered without any adverse effects.

## DISCUSSION

We present a case report demonstrating the feasibility and safety of rapid methadone titration in the ED to initiate MOUD. This case highlights the importance of methadone as a treatment option, particularly in the era of HPSOs, which have complicated OUD treatment. Methadone offers a crucial alternative to buprenorphine that mitigates the risk of precipitated withdrawal caused by buprenorphine and is associated with a lower risk of treatment discontinuation, particularly at the beginning of the treatment episode. In addition, the use of shared decision-making and clinical judgment to choose between buprenorphine and methadone, with careful consideration of the patient’s level of exposure to HPSOs, use of other substances, and co-morbidities, leads to improved MOUD adherence and outcomes.^5^,^12^,^13^

Traditional methadone protocols recommend titrating to a maximum dose of 30–40 mg on the first day, increasing by 10 mg daily to reach 60 mg by day three, and maintaining this dose for five days before making further adjustments.^7^ Such a conservative approach may not be feasible for patients using HPSOs, as reaching a therapeutic dose may take over two weeks, during which time withdrawal and cravings may be inadequately controlled, potentiating relapse and overdose.^9^ New federal guidelines allow for more flexible dosing protocols, including higher initial doses that enable rapid titration to therapeutic levels. These regulations state that the initial dose of methadone shall be individually determined with consideration of the types of opioids and other substances the patient is using, medical history, and severity of opioid withdrawal.^10^

Under these new guidelines, patients must be assessed for opioid exposure. We suggest that patients using HPSOs daily be considered to have high opioid exposure, with a treatment goal of titrating the methadone dose to 40–70 mg before ED discharge. In contrast, patients with lower opioid tolerance, such as those using only prescription opioids, only occasional opioids, or who have been exposed via contaminated stimulants (eg, cocaine, methamphetamine), need lower doses of methadone and should be initiated at 20–30 mg. Our case demonstrates how, with careful patient selection, rapid titration of methadone can be safely accomplished in the ED, an ideal setting for methadone initiation due to availability of cardiopulmonary monitoring, immediate access to medical interventions, and streamlined referrals to OTPs. Inpatient case reports have reported the successful induction of patients with severe OUD who were rapidly titrated on methadone starting at 30 mg with an additional 10 mg every three hours to a total of 60–70 mg on day one.^14^

Higher induction doses of methadone are not without risk. Methadone’s long half-life increases the risk of overdose, especially during the first two weeks of treatment. The most common complication associated with methadone administration is over-sedation, which typically does not require naloxone administration and can be effectively monitored in the ED.^15^ Given its complexity compared to buprenorphine, it is essential that emergency physicians are thoroughly trained in the proper administration and titration of methadone.[Fig f1-cpcem-9-188]

A limitation of our rapid methadone titration protocol is treatment time. Our patient was in the ED for 17 hours, although once the rapid methadone titration started, the patient was able to receive the maximum 70 mg dose and be discharged after a period of observation in under five hours. Additionally, it is imperative to have pre-existing agreements for rapid follow-up at a local OTP and a plan for methadone bridge dosing if the patient presents to the ED on a weekend or holiday.

## CONCLUSION

While buprenorphine initiation has become the ED standard for medication for opioid use disorder, the prevalence of high-potency synthetic opioids and the risk of precipitated withdrawal often necessitate treatment with methadone. In the era of HPSOs, effective methadone dosing requires rapid titration to achieve therapeutic levels quickly. We recommend considering rapid methadone titration in the ED to initiate MOUD for carefully selected patients with OUD and high opioid exposure, following shared decision-making and thorough assessment.

## Figures and Tables

**Figure f1-cpcem-9-188:**
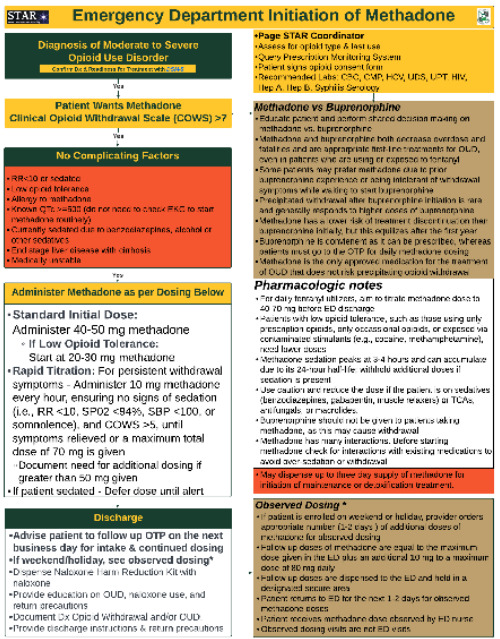
Emergency department rapid titration of methadone Start Treatment and Recovery pathway. *CBC*, complete blood count*; CMP*, comprehensive metabolic panel*; COWS*, Clinical Opiate Withdrawal Scale; *ED*, emergency department; *EKG*, electrocardiogram; *mg*, milligram; *HCV*, hepatitis C; *OTP*, outpatient treatment program; *OUD*, opioid use disorder; *RR*, respiratory rate; *SpO_2_*, oxygen saturation; *STAR*, Start Treatment and Recovery; TCA, tricyclic antidepressant; *UDS*, urine drug screening; *UPT*, urine pregnancy test.

**Table:** Patient vital signs and Clinical Opiate Withdrawal Scale scores during methadone titration.

Time Elapsed	RR (rpm)	HR (bpm)	BP (mm Hg)	SpO_2_% (Room air)	Clinical Opiate Withdrawal Scale	Amount of Methadone Administered
0:00	12	89	110/79	97	14	50 mg
1:25	16	77	115/84	100	10	10 mg
2:59	-	83	109/80	98	6	10 mg
4:48	15	71	107/65	98	6	-

*BP*, blood pressure; *bpm*, beats per minute; *HR*, heart rate; *mg*, milligram; *mm Hg*, millimeters of mercury; *rpm*, respirations per minute; *RR*, respiratory rate; *SpO2*, oxygen saturation.
